# The Effect of Online Chronic Disease Personas on Activation: Within-Subjects and Between-Groups Analyses

**DOI:** 10.2196/resprot.3392

**Published:** 2015-02-25

**Authors:** Catherine Devany Serio, Jason Hessing, Becky Reed, Christopher Hess, Janet Reis

**Affiliations:** ^1^HealthwiseBoise, IDUnited States; ^2^Boise State UniversityCollege of Health SciencesBoise, IDUnited States

**Keywords:** chronic disease, self-management, Internet, patient-centered care

## Abstract

**Background:**

Although self-management of chronic disease is important, engaging patients and increasing activation for self-care using online tools has proven difficult. Designing more tailored interventions through the application of condition-specific personas may be a way to increase engagement and patient activation. Personas are developed from extensive interviews with patients about their shared values and assumptions about their health. The resulting personas tailor the knowledge and skills necessary for self-care and guide selection of the self-management tools for a particular audience.

**Objective:**

Pre-post changes in self-reported levels of activation for self-management were analyzed for 11 chronic health personas developed for 4 prevalent chronic diseases.

**Methods:**

Personas were created from 20 to 25 hour-long nondirected interviews with consumers with a common, chronic disease (eg, diabetes). The interviews were transcribed and coded for behaviors, feelings, and beliefs using the principles of grounded theory. A second group of 398 adults with self-reported chronic disease were recruited for online testing of the personas and their impact on activation. The activation variables, based on an integrated theory of health behavior, were knowledge of a given health issue, perceived self-management skills, confidence in improving health, and intention to take action in managing health. Pre-post changes in activation were analyzed with a mixed design with 1 within-subjects factor (pre-post) and 1 between-group factor (persona) using a general linear model with repeated measures.

**Results:**

Sixteen pre-post changes for 4 measures of activation were analyzed. All but 2 of the within-subjects effects were statistically significant and all changes were in the direction of increased activation scores at posttest. Five significant differences between personas were observed, showing which personas performed better. Of low activation participants, 50% or more shifted to high activation across the 4 measures with minimal changes (≤5%) in the reverse direction.

**Conclusions:**

The majority of participants using a persona-tailored learning path reported high levels of satisfaction with their online user experience and increased levels of activation about their own health. In the body of work on patient activation, the current study adds to understanding of both short-term impact and the content of a brief, online intervention for engagement of specific groups in self-management.

## Introduction

Management of a chronic disease requires knowledge about that disease, skills for handling problems related to the disease, confidence in being able to execute those necessary skills, and intention to do so [1]. Without this requisite self-care, a patient’s condition often deteriorates, sometimes irreversibly [2]. Despite many efforts to ameliorate these conditions through enhanced patient self-management, the prevalence rates for diseases such as diabetes [3] and asthma [4] continue to increase while the rates for other diseases such as heart disease [5] and depression [6] have not declined as hoped. The overall profile of health points to the necessity of increasing patient activation with self-care as a first step in successful disease management.

The construct of activation is receiving more attention as a predictor of patient behavior. Recent cross-sectional surveys have found a range of measures of activation predictive of information seeking [7], health status [8-12], satisfaction with care and provider interaction [13,14], and self-management [11,15]. However, comparatively little work has been done on ways to increase activation, and all interventions have been some type of in-person instruction and/or coaching [15,16].

Personas generated from the Mental Models paradigm offer a form of tailored messaging which may boost activation. In this approach, personas are created through nondirected interviews with consumers to determine the user experience, common values, and assumptions about a given product [17]. In the domain of health and disease management, personas are a tool for understanding what a person who has identified with a given persona would do with their self-care, their expectations about the outcomes of treatment, and what additional information might be useful to them in making their decision to take action [18,19].

Personas may be particularly effective online in addressing different patient perspectives about self-management of chronic diseases [20,21]. Online material is readily accessible for many people, can be presented in a range of visual forms, and allows for iterative exploration at the convenience and interest of the user. Personas may add to the versatility and usefulness of online health education materials since they are tailored for specific audiences. Patients’ selection and reaction to personas may be of use to clinicians seeking to understand the perspectives and preferences of their patients.

This paper summarizes user feedback on 11 online personas developed for 4 prevalent chronic diseases with the aim of increasing activation. The effectiveness of the self-selected personas was assessed first with averaged pre-post changes on the 4 elements of activation. Effectiveness was also analyzed with a tally of the percentage of people with a low level of activation who moved toward a higher state and vice versa. In addition, aspects of user experience with their selected persona are also summarized. The following research questions were asked:

1. Are there changes in self-reported activation level when a consumer selects a health persona and receives persona-specific self-management information and tools?
2. Are there differences between persona groups in self-reported activation metrics of knowledge, skills, confidence, and intent to act?
3. Is there a shift in activation level from the lowest levels of activation at pretest to higher activation at posttest across the personas?

This paper provides the first empirical evidence of how use of a chosen persona affects level of activation in consumers with 1 of 4 chronic diseases. Thus, the results summarized here provide direction as to how a particular methodology might be used to engage and activate individuals with a chronic disease.

## Methods

### Overview

The description of the methods is divided into 2 main sections. The first section provides an overview of the process used to develop the chronic disease personas. This includes a summary of the recruitment process, interviews, coding of the interview content, and construction of the entire persona.

The second section presents details on the recruitment of the online users for evaluating the impact of the personas on personal activation for chronic disease self-care, a description of the user experience, a description of the scales used pre- and postreview of the personas, and the data analysis plan.

### Persona Development

#### Theory

The Mental Model methodology was used to construct a total of 11 personas related to the 4 courses on chronic disease. This method was developed by Indi Young, cofounder of the design agency Adaptive Path. The methodological approach creates the mental models of audiences by aggregating affinity themes of behaviors, beliefs, and feelings expressed by members of the target audience during nondirected interviews [17]. Applying a mental model to a program gives developers greater insight into the moods and motivations of their audience.

#### Patient Interviews

Creation of each persona began with completion of 20 to 25 nondirected interviews with consumers recruited from telephone-based recruitment services using a national database and publically published residential information. Interviewees consisted of geographically distributed American adults between the ages of 25 and 65 years with type 2 diabetes mellitus, depression, coronary artery disease (CAD), or asthma reported as their main chronic disease.

The interviews lasted on average 60 minutes per person and were conducted by 1 of 4 interviewers recently trained in the Mental Model methodology [11]. Interviewers avoided influencing the words used by interviewees by asking very open-ended questions such as “What is it like, day to day, to have asthma?” or “Walk me through a day of your life as you deal with asthma.” Interviewees were asked to elaborate on issues they brought up, especially regarding behaviors or tasks they said they did.

The interviews were transcribed and subsequently coded for behaviors, feelings, and beliefs using the principles of grounded theory [22]. Interview data were organized into patterns based on the conceptual similarity of the data, also known as affinity themes. Two coders independently reviewed each transcript and then met with at least 1 other more experienced coder to mediate any disagreements and facilitate reliable, consistent coding. Exemplars of statements expressing behaviors, feelings, and beliefs for a given chronic disease were discussed until consensus was achieved.

#### Persona Construction

An interdisciplinary team of physicians, a clinical psychologist, experts in user experience, and experts in health content reviewed all the information from primary and secondary sources to outline the characteristics of the personas. Primary source data were the transcribed interviews previously mentioned; secondary sources per persona included 10 to 20 qualitative studies that included actual patient quotes published in English-based, medically reviewed journals within the last 10 years. Based on the main objective of the persona, the team labeled the individual represented. For example, CAD personas were labeled “Getting Heart Smart,” “Staying Heart Healthy,” and “Putting Your Heart First.” Table 1 lists the elements common to all personas constructed for the study.

**Table 1:** Elements of all chronic disease personas.

Persona characteristic	Description
Emotional hook	The fundamental emotion through which this persona can be connected with
In short	A succinct summary of the main thrust of this persona
Actual quote	A representative quote meant to portray something this persona might say
How I feel / perception of depression / openness to treatment	These represent the different variables that together establish the nature of this persona
Description	A short narrative describing this persona
Unique tasks	The affinity themes from the mental modeling process that map to this persona
Learning objectives	The instructional goals and clinical objectives for this persona
Content needs	The content required to address the learning objectives of the specific persona
Global content needs	The content required to address common learning objectives that span all personas for a given condition

For each persona, 4 to 5 information modules drawn from the Healthwise knowledgebase, patient instructions, and care support pages were matched to the chronic disease and validated by medical content specialists and a medical writer (eg, the “Getting Heart Smart” modules included “Will I Be Okay?,” “What Happened?,” “What Can I Do?,” “Why Medicines Work,” and “Who Can Help?”). Although a standard set of core modules served as the starting point for each persona, these modules were then condensed or enhanced to fit the unique content and tone needs of each persona. The final module concluded with 1 scenario-based question asking the participant to practice applying the concepts they learned up to that point. A summary tab kept a running tally of the modules completed and in progress, with the participant’s response to the question signifying completion of that module.

The key distinguishing factors upon which the modules were tailored included behavioral variables such as locus of control, perception of risk, presence of symptoms, perception of condition, openness to treatment, confidence, and acceptance of diagnosis. The number of modules available to a persona differed depending on the respective learning objectives and the persona’s information density tolerance. In contrast to the practice of profile creation, the tailoring of these personas hinged primarily on behavioral variables instead of demographic characteristics. Table 2 provides a listing of the 11 personas with key elements for each.

**Table 2:** Chronic disease personas with descriptions of key elements.

Condition persona name		Emotional hook	Summary	Representative quote	Tasks
**Asthma**					
	Tune in to You: Minimizing Maria	I’m managing	Not that big of a problem	“Well it doesn’t bother me every day...It’s not chronic or anything like that.”	Feel so accustomed to living with asthma; it doesn’t bother me every day
	Take Control: Steady Eddy	I’m okay	It’s a problem but it’s reasonably controlled	“I had cats but I ended up finding a home for them because the allergies and asthma were pretty bad.”	Keep the dust down; rather not rely on my inhaler
	Be Your Best: Severe Shauna	I’m struggling	It’s a problem and I’m worried	“My only hope is just that I can get it better somehow.”	Feel I have to be really careful; go in if it’s really bad
**Coronary artery disease**					
	Getting Heart Smart: Anxious Andy	I’m scared	Scared and open	“I never thought of death as I do now. That’s disturbing to me. It crops up once in a while.”	Fear having another heart attack; I have to change in order to live
	Putting your Heart First: Complacent Casey	I’m fine	All quiet on the healthfront	“...considering it has been what 7 years ago now that I had open heart surgery...I think I am doing fine when it comes to my heart disease.”	Not top of mind when no longer experiencing symptoms; doubt meds
	Staying Heart Healthy: Backburner Bobby	I’m spent	Not now, I’ve got soooo much to deal with	“Heart disease doesn’t play a part in my life [right now] because I have bigger problems.”	Care for myself inconsistently, avoid thinking about CAD
**Depression**					
	Finding Your Way: Lost Linda	I’m lost	Tell me I can feel better	“I miss my old self.”	Feel I am failing; I was not able to see I was depressed; feel it’s a result of physical pain
	Breaking the Cycle: Not Again Nate	I’m worried	Not again	“I need help before my next depression episode. It hits harder every time.”	Fear the next episode; keep busy to distract myself; feel unnerved friends don’t understand
	Climbing Out: Stuck Stella	I’m stuck	This must be as good as it gets	“I’ve been this way for a very, very long time.”	Feel I have tried it all; get totally fed up; believe I will always be this way
**Type 2 diabetes**					
	Keeping it Simple: No Med Ned	I’m determined	Highly motivated to avoid meds and reverse the diagnosis	“I can overcome this on my own. I’m going to do everything I can to avoid medications.”	Control blood sugar through lifestyle changes; avoid meds
	Making a Change: Open Oscar	I’m willing	Living with but doesn’t prioritize or understand condition	“Whatever you say doc.”	Be compliant with the doctor’s plan; please the doctor

### Online Persona Testing

#### Sample

An open Internet-based survey was used to test the impact of the personas on activation for chronic disease self-care. Contact with potential survey participants was initiated by the Sample Network, an online marketing research company specializing in online sampling and recruitment. The announcement given subsequently was used to initially identify participants. Demographic characteristics were collected by the sample provider and were used to ensure a representative sampling. The announcement was:

The purpose of this study is to obtain consumer feedback on information on chronic diseases produced by a not-for-profit health education company.

No personal information will be collected.

Participation is voluntary. If you choose to participate please complete both the pre and post questionnaires as well as at least 1 lesson from the health course. You will receive an incentive for participation per your terms of participation in Sample Network.

The Web-based questionnaires and the health course lesson will take an average of 15 minutes to complete.

Membership in the Sample Network’s panels is incentive-based. Various methods are used to vet panel participants and ensure quality responses (eg, the frequency with which participants are allowed to respond to surveys is regulated to weed out professional survey takers). Data were collected April 20-26, 2012. Sample Network provided the necessary URL to their panel members. Sample Network prevents duplicate entries from the same IP address.

Anonymous survey responses were collected on the Healthwise survey platform and subsequently analyzed.

The average recruitment rate for the final convenience sample across the 4 chronic diseases was 13.99% (398/2843) as calculated by the number of people expressing interest in participation versus the number found to be eligible according to their self-report of a chronic disease and a targeted persona path (asthma: 15.1%, 98/651 potential respondents; CAD: 26.2%, 98/364 potential respondents; diabetes: 19.0%, 101/531 potential respondents; depression: 9.96%, 103/1034 potential respondents). Differences in recruitment rates across the chronic conditions were due to challenges in matching respondents with certain personas and ensuring an even distribution of respondents across personas. There was a completion rate of 100% for those respondents included in the final sample. Complete surveys were analyzed with no adjustments to the initial responses.

#### User Experience

Participants self-selected into a persona by choosing a path based on a path title, an image, and a path quote. Multimedia Appendix 1 provides an illustration of what participants with depression were presented with to make their choice of persona.

To ensure balanced feedback across all the learning paths within a course, an equal number of participants were recruited for each path. Approximately equal subgroup sample sizes were achieved by screening at the first stage of recruitment. Once a learning path was full, individuals who voted for that path and who would have qualified for that path based on their presurvey response were not allowed to be part of the sample. An average of 100 users were tested for each chronic disease course. There was no difference in completion rates across the 11 personas.

Respondents were required to spend at least 4 minutes completing a lesson before they were allowed to proceed to the postquestionnaire. Average time spent in the lesson was 13 minutes. Timeframe for elimination of too-brief responses was based on usability testing with prior users. Only 1 response to each question was allowed, and respondents could review their answers and make changes using the “back” button.

Construction of software allowed for switching personas within a given course. There were 44 of 398 (11.1% of total sample) who chose a different learning path or persona from the one they voted for at the time of recruitment into the survey. The “Keeping it Simple” persona for the type 2 diabetes course accounted for 25% of those who switched. Switching from the initially selected learning path occurred for 1 to 5 people across the other personas. For purposes of this analysis, all people responding to the activation measures were grouped into their final persona regardless of initially selected persona. Multimedia Appendix 2 provides a complete description of the 3 depression personas.

#### Scales

Activation is conceptualized here within the framework of an integrated theory of health behavior which brings together theories of self-efficacy, stages of change, health beliefs, and intention to change [23,24]. The integrated theory incorporates elements of decision making predictive of a wide range of behaviors. The accepted measures are knowledge of a given health issue, perceived skills in making personal health better, confidence in being able to make personal health better, and intention to undertake changes in behavior for improvement of personal health (for purposes of this study, intention to act was focused on the week directly after participation in the study).

Participants were asked to assess their standing on the 4 elements of activation (knowledge, skill, confidence, and intention) on a 5-point Likert scale (1=strongly disagree to 5=strongly agree) at the beginning and end of the online course. These scales were taken from a previous study conducted with decision aids [25]. Participants were also asked to rate the course on a 6-point Likert scale according to the following descriptors: clear, helpful, trustworthy, taught something, built confidence, motivated to act, and worth sharing with others. Last, participants were asked at the conclusion of the course, using a 5-point Likert scale, to assess the degree to which they learned some new information that will be useful in their day-to-day life, whether the course gave them a new idea about something to try, and whether they were confident they could use something they learned from their course.

Following the example of Heller et al’s analysis of patient activation [13], low-activated respondents were classified as giving a response of 1, 2, or 3 to each of the 4 measures of activation. Scores of 4 and 5 designated activated patients. Surveys with complete responses were analyzed with no adjustments for the responses.

#### Data Analysis

Data were analyzed with a mixed design with 1 within-subjects factor (pre-post) and 1 between-group factor (persona). The general linear model with repeated measures in SPSS version 21 (IBM Corp, Armonk, NY, USA) was used for the analysis [26]. Since the pre-post dependent measures were significantly correlated with each other (*P*=.05), the multivariate results for Pillai’s trace (V) and related statistics are reported [27]. The assumption of equality of covariance matrices of the dependent measures was tested with Box’s test of equality of covariance matrices. In no instance was a significant difference (*P*<.001) for these observed covariance matrices found, thus meeting a key assumption of homogeneity of variance-covariance matrices [28]. Post hoc comparisons for significant between-subjects main effects were analyzed with the Bonferroni test [29]. Pearson’s chi-square test of association was used to analyze relationships between pre and post measures of activation and aspects of user experience.

### Participants

All participants agreed to a standard statement about personal privacy and use of their responses for research purposes. Consent was obtained a second time with users responding to the question, “Do you agree to the use of your survey responses as noted above?”

## Results

A total of 398 participants were recruited (249 women and 149 men). In all, 86.7% (345/398) of the participants were white/non-Hispanic; 7.0% (28/398) of the sample was African-American, and the rest were Native American, Asian, or multiracial (25/398). Of the sample, 89.9% (358/398) had completed at least some college coursework. Overall, participants tended to be middle-age and older with 5.0% (20/398) in the 18 to 24 age range, 15.1% (60/398) in the 25 to 34 age range, 39.9% (159/398) in the 35 to 54 age range, and 39.9% (159/398) in the 55-years-and-older category.

Potential associations between the sociodemographic variables and course selected were of interest for understanding the user experience. There was a significant association between age and course used (χ^2^_15_=72.5, *P*<.001), with CAD and diabetes course participants being older (51.5%, 52/101 and 64.2%, 61/95 were 55 years or older, respectively) as compared to participants in the other courses.

There was also a significant association between gender and course used (χ^2^_6_=47.8, *P*<.001). Women were more likely to participate in the asthma course (female: 79.6%, 82/103; male: 19.4%, 20/103), and the depression course (female: 76%, 75/99; male: 24%, 24/99). Men were more likely to participate in the CAD course (female: 38%, 36/95; male: 61%, 58/95). Participation in the diabetes course was approximately equal (female: 54.5%, 56/101; male: 43.6%, 44/101).

For the final rating, 80% (318/398) of respondents rated their course positively (strongly agree and agree) in terms of clarity, helpfulness, trustworthiness, teaching something new, worth sharing, and increased confidence. Participants in the depression course were less likely to feel the course had taught them something (χ^2^_12_=22.3, *P*=.03) or that they were confident they could use something they had learned (χ^2^_15_=24.4, *P*=.05).

Tables 3-6 present the pre-post activation scores by tailored learning path personas for each of the 4 chronic disease courses. Each activation measure was identified as a repeated pre-post dependent variable and the personas were identified as the between-subject factor. Significant within-subject multivariate results for Pillai’s trace are reported along with the associated *F* statistic and degrees of freedom. Values for partial eta squared are presented as a measure of effect size; in this case, the proportion of variance explained that is not explained by other variables.

**Table 3:** Results of general linear model with repeated measures analyses of chronic health disease personas: asthma.

Activation metrics		Personas, mean (SD)						V	*F* (*df*)	*P*	η^2^_partial_
		Be Your Best (Severe Shauna) n=32		Take Control (Steady Eddy) n=32		Tune in to You (Minimizing Maria) n=32					
		Pre	Post	Pre	Post	Pre	Post				
**Knowledge**											
	Within	3.88 (1.19)	4.41 (0.56)	3.84 (1.11)	4.16 (0.95)	4.28 (0.68)	4.44 (0.56)		11.14 (1,93)	.001	0.11
	Between							0.11	1.96 (1,3)	.15	
**Skills**											
	Within	3.75 (1.24)	4.41 (0.67)	3.75 (1.08)	4.06 (0.91)	4.00 (1.05)	4.38 (0.71)		16.05 (1,93)	<.001	0.15
	Between							0.15	0.770 (1,3)	.51	
**Confidence**											
	Within	3.88 (1.29)	4.41 (0.67)	3.64 (1.14)	4.03 (1.05)	3.94 (1.08)	4.28 (0.73)		17.16 (1,94)	.01	0.15
	Between							0.15	1.43 (1,3)	.24	
**Action**											
	Within	3.68 (1.42)	4.19 (0.98)	4.23 (0.71)	4.17 (1.02)	3.19 (1.14)	3.61 (1.17)		17.16 (1,94)	.001	0.15
	Between							0.15	2.24 (1,3)		

**Table 4:** Results of general linear model with repeated measures analyses of chronic health disease personas: coronary artery disease.

Activation metrics		Personas, mean (SD)						V	*F* (*df*)	*P*	η^2^_partial_
		Getting Heart Smart (Anxious Andy) n=33		Putting Your Heart First (Complacent Casey) n=26		Staying Heart Healthy (Backburner Bobby) n=32					
		Pre	Post	Pre	Post	Pre	Post				
**Knowledge**											
	Within	4.24 (1.12)	4.48 (0.62)	4.08 (0.80)	4.27 (0.67)	4.38 (0.75)	4.50 (0.62)		3.29 (1,89)	.07	
	Between							0.04	1.37 (1,3)	.26	
**Skills**											
	Within	4.00 (1.10)	4.52 (0.57)	3.73 (1.00)	4.04 (0.60)	4.39 (0.90)	4.42 (0.61)		7.90 (1,89)	.006	0.08
	Between							0.08	4.67 (1,3)	.01	0.09
**Confidence**											
	Within	4.03 (0.95)	4.42 (0.80)	3.42 (0.90)	3.92 (0.94)	4.23 (0.99)	4.42 (0.72)		9.68 (1,89)	.002	0.10
	Between							0.10	7.09 (1,3)	.003	0.14
**Action**											
	Within	3.68 (1.42)	4.19 (0.98)	4.26 (0.71)	4.17 (1.02)	3.19 (1.14)	3.61 (1.17)		13.43 (1,89)	<.001	0.14
	Between							0.15	1.93 (1,3)	.15	

**Table 5:** Results of general linear model with repeated measures analyses of chronic health disease personas: depression.

Activation metrics		Personas, mean (SD)						V	*F* (*df*)	*P*	η^2^_partial_
		Breaking the Cycle (Not Again Nate) n=33		Climbing Out (Stuck Stella) n=32		Finding Your Way (Lost Linda) n=32					
		Pre	Post	Pre	Post	Pre	Post				
**Knowledge**											
	Within	3.88 (0.96)	4.15 (0.67)	3.94 (0.80)	4.13 (0.83)	4.13 (1.01)	4.16 (0.68)		3.12 (1,93)	.08	
	Between							0.03	0.308 (1,3)	.74	
**Skills**											
	Within	3.70 (0.92)	4.00 (0.79)	3.45 (1.03)	3.77 (1.23)	4.09 (0.96)	4.09 (0.69)		4.53 (1,93)	.04	0.05
	Between							0.05	2.72 (1,3)	.07	
**Confidence**											
	Within	3.73 (1.01)	3.97 (0.77)	3.30 (1.21)	3.48 (1.35)	4.13 (0.83)	4.25 (0.67)		4.69 (1,95)	.03	0.05
	Between							0.05	6.17 (1,3)	.003	0.12
**Action**											
	Within	3.79 (0.86)	4.15 (0.71)	3.33 (1.19)	3.67 (1.24)	4.09 (0.78)	4.19 (0.69)		11.61 (1,95)	.001	0.11
	Between							0.11	4.87 (1,3)	.01	0.09

**Table 6:** Results of general linear model with repeated measures analyses of chronic health disease personas: type 2 diabetes.

Activation Metrics		Personas, mean (SD)				V	*F* (*df*)	*P*	η^2^_partial_
		Keeping it Simple (No Med Ned) n=48		Making a Change (Open Oscar) n=49					
		Pre	Post	Pre	Post				
**Knowledge**									
	Within	3.90 (1.10)	4.17 (0.72)	4.27 (0.88)	4.47 (0.58)		5.44 (1,95)	.05	0.05
	Between					0.05	3.96 (1,2)	.22	
**Skills**									
	Within	3.66 (1.17)	4.20 (0.76)	4.18 (0.88)	4.43 (0.61)		13.76 (1,97)	<.001	0.12
	Between					0.12	7.02 (1,2)	.07	0.07
**Confidence**									
	Within	3.59 (1.19)	4.04 (0.96)	4.14 (0.88)	4.44 (0.68)		14.23 (1,97)	<.001	0.13
	Between					0.14	8.63 (1,27)	.004	0.08
**Action**									
	Within	3.49 (1.16)	4.08 (0.91)	3.98 (0.86)	4.24 (0.82)		23.35 (1,93)	<.001	0.20
	Between					0.20	3.46 (1,2)	.07	

All but 2 of the within-subjects effects were significant at the .04 level or less on all pre-post activation measures. No pre-post differences in knowledge were observed for the depression course or the CAD course. Across personas within the 4 courses on the 4 pre-post activation measures, all change was in the direction of increased scores at posttest. Five between-subjects effects were significant at the .01 level or less. No statistically significant interactions were observed between the within-subject factor and the personas as a between-subject factor.

Five post hoc contrasts were observed to be significant at the .01 level or less for the 5 between-subjects effects for which these contrasts could be calculated. (Diabetes had only 2 learning paths and, therefore, contrasts could not be calculated.) Within CAD, “Putting Your Heart First” was the least positively assessed tailored content learning path and “Staying Heart Healthy” was the most positively assessed for confidence (mean difference=0.55, *P*=.009) and skills (mean difference=0.52, *P*=.01). “Putting Your Heart First” was also least positively assessed for confidence as compared to “Getting Heart Smart” (mean difference=0.65, *P*=.002). The tailored content learning paths for the depression course were mixed, with “Climbing Out” more negatively assessed for confidence (mean difference=0.79, *P*=.002) and “Breaking the Cycle” more negatively assessed for action as compared to “Finding Your Way” (mean difference=0.64, *P*=.01).

The same mixed within-subjects and between-subjects design was used to analyze pre-post differences in knowledge, skills, confidence, and intention with course status (completed or in progress) as a 2-level between-subjects factor. All within-subjects effects were found to be significant at the *P*=.001 level or less (pre-post knowledge: V=0.04, *F*_1,396_=24.76, *P*=.001, η^2^_partial_=0.04; pre-post skills: V=0.06, *F*_1,396_=40.34, *P*=.001, η^2^_partial_=0.06; pre-post confidence: V=0.07, *F*_1,396_=43.05, *P*=.001, η^2^_partial_=0.07; pre-post intentions: V=0.07, *F*_1,396_=43.60, *P*=.001, η^2^_partial_=0.07). There were no significant between-subjects effects.

Overall changes in pre-post levels of activation were first examined for shifts in the lowest levels of activation (1, 2, and 3) at pretest to high activation at posttest (4 or 5). Across the 4 courses at pretest, a total 84 of 398 respondents (21.1%) were classified as being low activation on knowledge, 110 (27.6%) were classified as being low activation on skills, 123 (30.9%) were classified as being low activation on confidence, and 137 (34.4%) were classified as being low activation on intention to act. All chi-square associations between pre and post measures of activation were significant at *P*<.001.

Tallying the number of participants shifting from the lowest levels of activation to the highest levels at posttest across the 4 chronic conditions, 76% (66/87) of low activation for knowledge changed to high activation, 64.2% (70/109) of low activation for skills changed to high activation, 56.1% (69/123) of low activation for confidence changed to high activation, and 51.4% (71/138) of low intention to act changed to high activation. Conversely, the percentage of respondents with the highest level of activation (5) decreased to the lowest levels (1, 2, 3) at posttest by 5% for knowledge, 3% for skills, 5% for confidence, and 8% for intention to act.

In total, 45.9% (183/398) of the total sample provided written, free-field details on what they planned to change short-term regarding their health. Exercise was the most frequently mentioned change (67.1%, 267/398 of write-in responses), followed by changes in diet toward healthier eating (41.9%, 167/398 of write-in responses). Additionally, individuals with asthma stated they would pay more attention to triggers and medicines (19%, 18/96 of all asthmatics) and 26% (25/97) of those with depression wrote in that they would try and relax more, be less stressed, and/or be more positive.

## Discussion

### Principal Findings

The results of this study document a generally positive reaction to online chronic disease personas constructed from feedback from individuals with 1 of 4 chronic diseases who had selected a persona with whom they felt affinity. The majority of participants using a chosen learning path reported high levels of satisfaction with their user experience and increased levels of activation with regards to their own health. The participants who selected personas within the depression course reported the smallest changes in activation overall. Within a chronic disease, “Putting Your Heart First” was related to lower activation levels at posttest as compared with the other 2 CAD-tailored content personas. The calculated effect sizes ranged from 0.05 to 0.15, bracketing the overall similar small effect size of 0.074 reported by Noar et al [30] for tailored print messages and the posttest effect of 0.14 reported for Web-delivered behavior change interventions [31].The implications of the participants’ experiences are considered after acknowledgment of key methodological limitations.

In the body of work on activation, this study adds to understanding of both short-term impact and the content of a brief, online intervention. Descriptions of the actual elements of interventions for increasing activation are sparse [15,32]. Therefore, a contribution of this study is more details on the structure and content of the personas used to address a person’s level of activation. A second contribution arises from use of the Internet to deliver the activation materials to individuals with a chronic disease. Other efforts at activation have relied on various forms of in-person, one-on-one health coaching, telephone contacts, and/or information campaigns [16,33]. The Internet offers another portal convenient for many to use and flexible in delivery [34].

### Limitations

A major limitation is reliance on self-reports of both health status and intention to change their interactions with their physician about management of the participant’s chronic condition. Additional research is required to determine if individual behavior actually does change and, if so, which specific behaviors were altered and for how long. This would be especially important given that the sample recruited through the online survey service for this study was skewed toward a higher-educated, primarily white/non-Hispanic group.

Understanding of the impact of the personas would be further enhanced with inclusion of a comparison group formed through random assignment. A comparison group was not included here because of limitations of resources. Inclusion of a comparison group would allow for more direct testing of the content of the persona on patient activation against standard patient educational material. This, in turn, would provide a gauge of the advantages gained through a persona and the tailored material contained therein.

Additionally, the sample sizes within a given tailored content learning path were relatively small. However, Pillai’s trace is considered the most reliable of the multivariate measures and offers the greatest protection against type I errors with small sample sizes. [27] The Bonferroni method controls the overall type I error and is the method commonly used for all pairwise comparisons tests. MANOVA is robust to violations of multivariate normality and to violations of homogeneity of variance-covariance matrices if groups are of nearly equal size (n of the largest group is no more than 1.5 times the n of the smallest group).

In terms of completeness of theory, this study assessed 4 of the 8 variables deemed important for behavioral change from 1 integrated theoretical paradigm [24,35]. Further research would be needed to determine the contribution of the environment, pros and cons, social norms, and consistency with self-image. Also, a potential limitation is use of single items for determination of knowledge, confidence, skills, and intention. However, other studies of the role of activation in self-care have relied on fewer and less theoretically based measures [7,13].

### Clinical Implications

Health systems and individual clinicians are in the midst of a tsunami of change given the transition to Accountable Care Organizations and other forces within the health care system [36,37]. Additionally, health systems are transitioning to population-based care and aiming to reach increasing number of patients in more automated ways. We believe in order to engage populations of patients in their own self-management, automated interventions must be designed from the patient perspective. Personas founded on the user’s emotions, thoughts, and actions put the patient at the center of the experience and create resonance and engagement. Once patients are engaged, use of automated self-management programs puts the best behavior change science right in the palm (ie, cell phone) of the patient’s hands. These interventions may act as clinician extenders with the behaviorally designed self-management tools helping the patient know how to achieve the recommended goals in their day-to-day life.

## Multimedia Appendix 1

Introduction to depression personas.

## Multimedia Appendix 2

Overview of depression personas.

## Abbreviations

CAD: coronary artery disease
