# Supplementary material for: The Effect of Online Chronic Disease Personas on Activation: Within-Subjects and Between-Groups Analyses
Source: JMIR Res Protoc. 2015 Feb 25;4(1):e20. doi: 10.2196/resprot.3392 (PMC4376159; doi:10.2196/resprot.3392)
Supplement: Supplementary file 1 [file resprot_v4i1e20_app1.pdf]

Choose the path that fits you best.

Finding Your Way

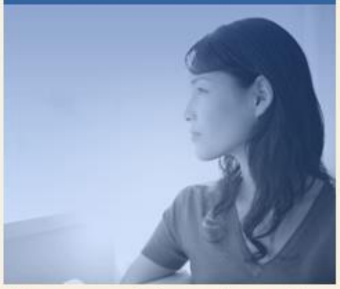

"I was stunned when my doctor said this was depression. Now I'm afraid I'll never see my old awesome self again. Some days I just feel hopeless."

Start »

Breaking the Cycle

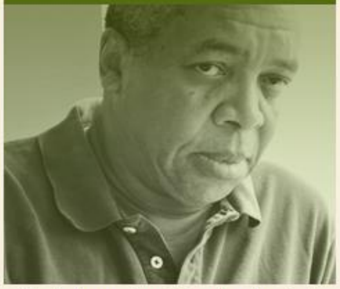

"I had depression before, but I worked through it. I thought that was the end of it. But now it's back, and that makes me mad. I'm sad, frustrated, irritated—sometimes all at once."

Start »

Climbing Out

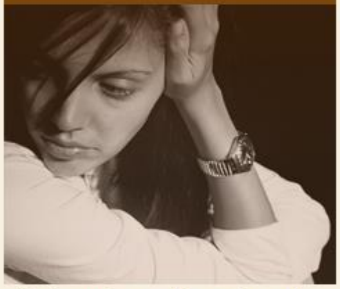

"I've been depressed for as long as I can remember. The medicine has helped some, but I still just feel so stuck in place. I guess I'm always going to be like this."

Start »
