# Supplementary material for: The Effect of Online Chronic Disease Personas on Activation: Within-Subjects and Between-Groups Analyses
Source: JMIR Res Protoc. 2015 Feb 25;4(1):e20. doi: 10.2196/resprot.3392 (PMC4376159; doi:10.2196/resprot.3392)
Supplement: Supplementary file 2 [file resprot_v4i1e20_app2.pdf]

L2E Depression Personas – ver 1.2

Based on Depression Secondary Research Model
Created by L2E Depression Team
Last Updated: 7/27/2011

	1 Finding Your Way	2 Breaking the Cycle	3 Climbing Out
	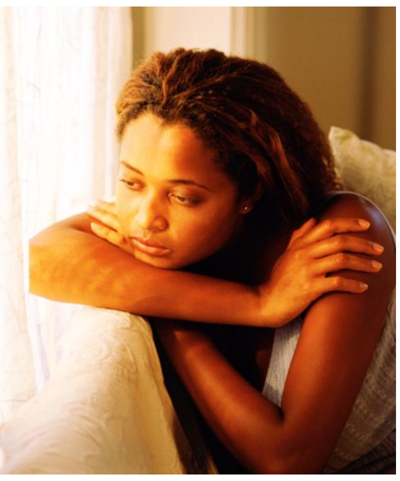	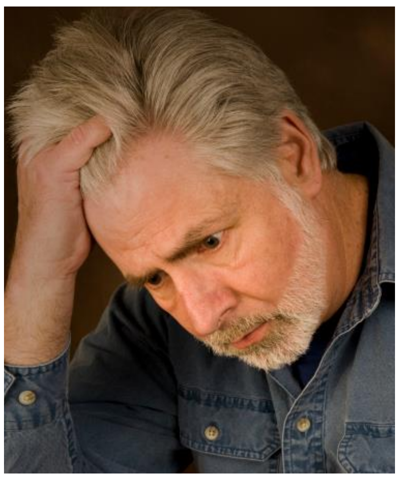	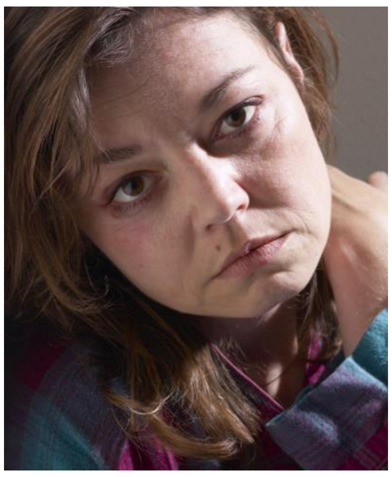
**Emotional Hook**	I'm lost.	I'm worried.	I'm stuck.
**In Short**	Tell me I can feel better	Not again	This must be as good as it gets
**Actual Quote**	"I miss my old self."	"I need help before my next depression episode. It hits harder every time."	"I've been this way for a very, very long time."
**How I Feel**	Struggling; something is off; sad or irritable; bewildered; flat	Sad or irritable; worried about falling back into a depressed state	Given up
**Perception of Depression**	Inexperienced / Unsure	Accepting & Experienced	Resigned
**Openness to Treatment**	Mostly open to treatment but with preferences	Open to treatment but with experience and with preferences	Lack of confidence in treatment
**Description**	Coming to terms with the diagnosis for the first time; wants to feel better; accepting of help	Been there done that depression thing; could go on and off meds.	Severe chronic; high risk; may perceive depression as a character flaw; emotional suffering is recognized and embraced; being treated but undertreated—need to step up meds.
**Unique Tasks**	Feel I am failing; I was not able to see I was depressed; Feel it's a result of physical pain	Fear the next episode; Keep busy to distract myself; Feel unnerved friends don't understand	Feel I have tried it all; Get totally fed up; Believe I will always be this way
**Learning Objectives**	FROM PAIN TO PLAN<br>↑acceptance, ↑skills, ↑knowledge, ↑hope, ↓uncertainty, ↑confidence in self	FROM CONCERN TO CONTROL<br>↑skills, ↓fear, ↔knowledge, ↑confidence in treatment	FROM MOLASSES TO MOMENTUM<br>↑motivation, ↑confidence (in ability to feel better), ↑confidence in treatment, ↑perception / awareness of depression as a disease
**Content Needs**	This is how treatment works; stigma strong; depression 101; how to talk about this with others	Draw on prior experience with past treatment; dealing with fears and the how to's of chronic management	Multiple meds; dosage; expect to feel better; go get more treatment; baby steps; stand up (not run)

Global Content Needs Putting on the mask; dealing with the stigma; feeling isolated; resistance to meds and to counseling
